# Supplementary material for: The newly-arisen Devil facial tumour disease 2 (DFT2) reveals a mechanism for the emergence of a contagious cancer
Source: eLife. 2018 Aug 14;7:e35314. doi: 10.7554/eLife.35314 (PMC6092122; doi:10.7554/eLife.35314)
Supplement: Supplementary file 1. — Details of the tumour and host tissue samples, the date the samples were collected from the animal and the location of the animal when it was trapped. *As described previously (Pye et al., 2016b). [file elife-35314-supp1.docx]

| **Animal** | **Alternative name** | **Date** | **Location** | **Tissue samples** |
| --- | --- | --- | --- | --- |
| TD202* | Red Velvet, RV | March 2014 | Cygnet, Tasmania | Tumour 1 |
|  |  |  |  | Spleen |
| TD203 * | Snug, SN | October 2014 | Snug, Tasmania | Tumour 2 |
|  |  |  |  | Spleen |
| TD549 |  | December 2015 | Woodbridge, Tasmania | Tumour 1 |
| TD818 | Teufel | January 2016 | Snug, Tasmania | Tumour 1 |
|  |  |  |  | Spleen |
| TD812 | Margaret | January 2016 | Margate, Tasmania | Tumour 1 |
| TD523 | 638T1 | June 2015 | Snug, Tasmania | Tumour 1 |
| TD547 | 807T1 | October 2015 | Cygnet, Tasmania | Tumour 1 |
