# Supplementary material for: The newly-arisen Devil facial tumour disease 2 (DFT2) reveals a mechanism for the emergence of a contagious cancer
Source: eLife. 2018 Aug 14;7:e35314. doi: 10.7554/eLife.35314 (PMC6092122; doi:10.7554/eLife.35314)
Supplement: Supplementary file 2. [file elife-35314-supp2.docx]

| **Gene amplified** | **Primer set** | **Sequence** | **Product size (bp)** | **Annealing temp.** |
| --- | --- | --- | --- | --- |
| Saha-UC (SahaI*27) full length | Saha349(F)  Saha350(R) | 5’ATACATATGTCTCACTCCTTGAGGTACTTCGACACCGCC3’  5’ATATCTCGAGTTTGGCTGTCAGAGAGACATCTGACCC3’ | 1000bp | 60 °C |
| Saha-UK full length | Saha335(F)  Saha351(R) | 5’ATACATATGGTTCATTCTCTGAGGTATTTCCAAACTTCC3’  5’ATATCTCGAGTTTGGCTGTCAGAGAGACATCTGATCC3’ | 1000bp | 60 °C |
| RPL13A | Saha118 (F)  Saha119 (R) | 5’CCCCACAAGACCAAGCGAGGC3’  5’ACAGCCTGGTATTTCCAGCCA3’ | 300bp | 60 °C |
| MHC class I classical *Saha-UA, -UB,* and *–UC* | Primer set 1 | 5’CCGTGGGCTACGTGGACGA3’  5’GTCGTAGGCGAACTGAAG3’ | 296bp | 60 °C |
| MHC class I non-classical *Saha-UK* | Primer set 2 | 5’TGGTTGGACAAGAGTAA3’  5’CCTCAGGAAGATCCAGTCGTAAGTC3’ | 190bp | 60 °C |
| MHC class I non-classical *Saha-UD* | Primer set 3 | 5’ATGGAGAATGTGGACCGGGAC3’  5’TGAGTTCACTGCCTCATTCACT3’ | 275bp | 59 °C |
| β_2_m | Saha22(F)  Saha23(R) | 5’TGTGCATCCTTCCCTACCTGGAGG3’  5’CATTGTTGAAAGACAGATCGGACCGC3’ | 300bp | 60 °C |
