# Supplementary material for: The newly-arisen Devil facial tumour disease 2 (DFT2) reveals a mechanism for the emergence of a contagious cancer
Source: eLife. 2018 Aug 14;7:e35314. doi: 10.7554/eLife.35314 (PMC6092122; doi:10.7554/eLife.35314)
Supplement: Supplementary file 3. [file elife-35314-supp3.docx]

| **Antibody** | **Clone or Cat Number** | **Supplier (where applicable)** |
| --- | --- | --- |
| Classical MHC class I Saha-UA, -UB and -UC | α-UA/UB/UC_15-25-18 | NA (this paper) |
| Non-classical MHC class I Saha-UK | α-UK_15-29-1 | NA (this paper) |
| IgG1 Isotype control from murine myeloma | M5284 | Sigma |
| IgG2b Isotype control from murine myeloma | M5534 | Sigma |
| β_2_m | Saha-13-34-48 | Pye et al, 2016 |
| CD3 | A0452 | Dako |
