# Supplementary material for: The newly-arisen Devil facial tumour disease 2 (DFT2) reveals a mechanism for the emergence of a contagious cancer
Source: eLife. 2018 Aug 14;7:e35314. doi: 10.7554/eLife.35314 (PMC6092122; doi:10.7554/eLife.35314)
Supplement: Supplementary file 4. [file elife-35314-supp4.docx]

| **PCR Reagents** | | **PCR Reaction Conditions** | | | |
| --- | --- | --- | --- | --- | --- |
| Reagent | Final concentration | Cycle element | Temp (°C) | Time (sec) | Number of cycles |
| cDNA | 500 ng | Initial denaturation | 98 | 120 | 1 |
| DNA polymerase (Thermofischer *Phusion*) | 0.5 U | Denaturing | 98 | 15 | 32 |
| Primers | 0.6 μM | Annealing | Primer anneal temperatures found in Table S3 | 30 |  |
| dNTPs | 200 μM |  |  |  |  |
| Phusion High Fidelity buffer | 1X | Elongation | 72 | 30 |  |
| ddH_2_O | To total volume of 25 μl | Final elongation | 72 | 300 | 1 |
